# Vascular endothelial growth factor: a key factor in the onset and treatment of depression

**DOI:** 10.3389/fncel.2025.1645437

**Published:** 2025-09-02

**Authors:** Jing Wang, Fanhao Meng, Long Wang, Zeguang Li

**Affiliations:** ^1^The Graduate School, Heilongjiang University of Chinese Medicine, Harbin, Heilongjiang, China; ^2^The First Affiliated Hospital of Heilongjiang University of Chinese Medicine, Harbin, China

**Keywords:** vascular endothelial growth factor, brain-derived neurotrophic factor, depression, blood-brain barrier, ketamine

## Abstract

**Background:**

Major depressive disorder (MDD) is a common chronic psychiatric disorder that affects individuals of all ages worldwide, causing significant impairment to patients’ physical and mental health as well as social functioning. Vascular endothelial growth factor (VEGF), traditionally recognized as a regulator of angiogenesis and vascular permeability, has been identified in recent studies to possess neurotrophic and neuroprotective potential in the central nervous system (CNS) and is implicated in the pathological processes of MDD.

**Aim:**

To systematically elaborate on the role of VEGF in the pathological mechanisms of MDD and its potential as a target for antidepressant therapy.

**Key findings:**

Through interactions with its receptors (VEGFR1, VEGFR2, and VEGFR3), VEGF regulates critical pathways such as gene expression, blood-brain barrier (BBB) function, and brain-derived neurotrophic factor (BDNF), thereby establishing physiological and pathological associations with MDD. Its signaling pathway serves as a core target for various antidepressant treatments, including conventional antidepressants, ketamine, electroconvulsive therapy (ECT), repetitive transcranial magnetic stimulation (rTMS), and resolvins. Short-term upregulation of central VEGF may exert antidepressant effects by promoting the benign remodeling of neurovascular networks, and its subsequent return to baseline levels during treatment can avoid BBB damage, providing novel insights for the management of rapid-onset and treatment-resistant depression.

**Conclusion:**

Vascular endothelial growth factor holds significant importance in the pathology and treatment of MDD. In-depth exploration of its regulatory mechanisms may provide a basis for the development of novel antidepressant therapies.

## 1 Introduction

Major depressive disorder (MDD) is a common mental disorder with a chronic course, characterized primarily by persistent and uncontrollable mood disturbances and cognitive impairments. It is often accompanied by fatigue, diminished interest, loss of pleasure, and suicidal ideation, affecting over 300 million people worldwide (GBD 2021 Diseases and Injuries Collaborators, 2024; Meng and Wang, 2023). The World Health Organization notes that MDD is one of the leading causes of mental and physical disability globally. Over the past half-century, the number of people with depression worldwide has increased by nearly 50% (Liu et al., 2020); notably, 28% of new cases globally emerged in 2020 alone, with a marked rise in prevalence in countries severely affected by COVID-19, where females and young people are the most impacted groups (COVID-19 Mental Disorders Collaborators, 2021). According to statistics published in 2023, the economic costs attributed to MDD in the United States alone have increased by 48% compared to previous periods (Greenberg et al., 2023). MDD has thus become a major threat to global health, with its economic and societal impacts continuing to intensify (Monroe and Harkness, 2022).

Many complex pathological processes are involved in the development and progression of MDD, such as genes, inflammation, mitochondrial dysfunction, oxidative stress, and the HPA axis (Dean and Keshavan, 2017; Fries et al., 2023). Neurotrophic factors play a key role in normal brain development and function, and the neurotrophic hypothesis of MDD focuses on fluctuations in neurotrophic factors within the center with changes in depressive symptoms, suggesting that impaired neurotrophic support is a key mechanism for MDD-associated neurological activity and brain-related changes (Kishi et al., 2017). Recent data suggest that levels of the key protein Vascular Endothelial Growth Factor (VEGF) may be an important marker of MDD and that its alteration is necessary to promote the pathologic progression of MDD (Annam et al., 2024). In turn, traditional antidepressant drugs are required to alleviate depressive symptoms with the help of VEGF signaling to exert their pharmacological effects (Warner-Schmidt and Duman, 2008). Thus, VEGF is a key player in the pathogenesis, amelioration, and treatment of depression, and the detection and targeting of VEGF may provide new ideas for the clinical prevention and treatment of MDD.

## 2 Mechanism of action of VEGF

### 2.1 VEGF and the receptor family

Vascular endothelial growth factor was initially discovered and named for its roles in regulating vascular permeability and promoting angiogenesis (Dvorak, 2006). As a key signaling molecule, VEGF is widely expressed throughout the body; it is not only present in neurons, astrocytes, and vascular endothelial cells within the central nervous system (Jais et al., 2016) but also produced by various peripheral cell types, including epithelial cells, smooth muscle cells, fibroblasts, macrophages, tumor cells, and retinal pigment epithelial cells (Preusser et al., 2012; Wang et al., 2025; Wartiovaara et al., 1998). This broad cellular source underpins VEGF’s central role in systemic physiological and pathological processes. Its classical functions include significantly increasing vascular permeability, facilitating extracellular matrix remodeling, stimulating the migration and proliferation of vascular endothelial cells, and ultimately driving the formation of new blood vessels (Connolly et al., 1989; Ferrara and Henzel, 1989; Senger et al., 1983).

Within the central nervous system (CNS), in addition to its involvement in regulating blood-brain barrier (BBB) function and angiogenesis, VEGF exhibits important neurotrophic and neuroprotective properties (Rosenstein et al., 2003). Through interactions with specific receptors on endothelial cells, neurons, and glial cells, it activates downstream signaling pathways and influences gene expression. These actions collectively promote neuronal survival, the proliferation of neural progenitor cells, and the self-renewal of embryonic and adult neural stem cells, thereby exerting a significant impact on neuronal plasticity and neurogenesis (Shen et al., 2004; Tillo et al., 2012).

**FIGURE 1:**
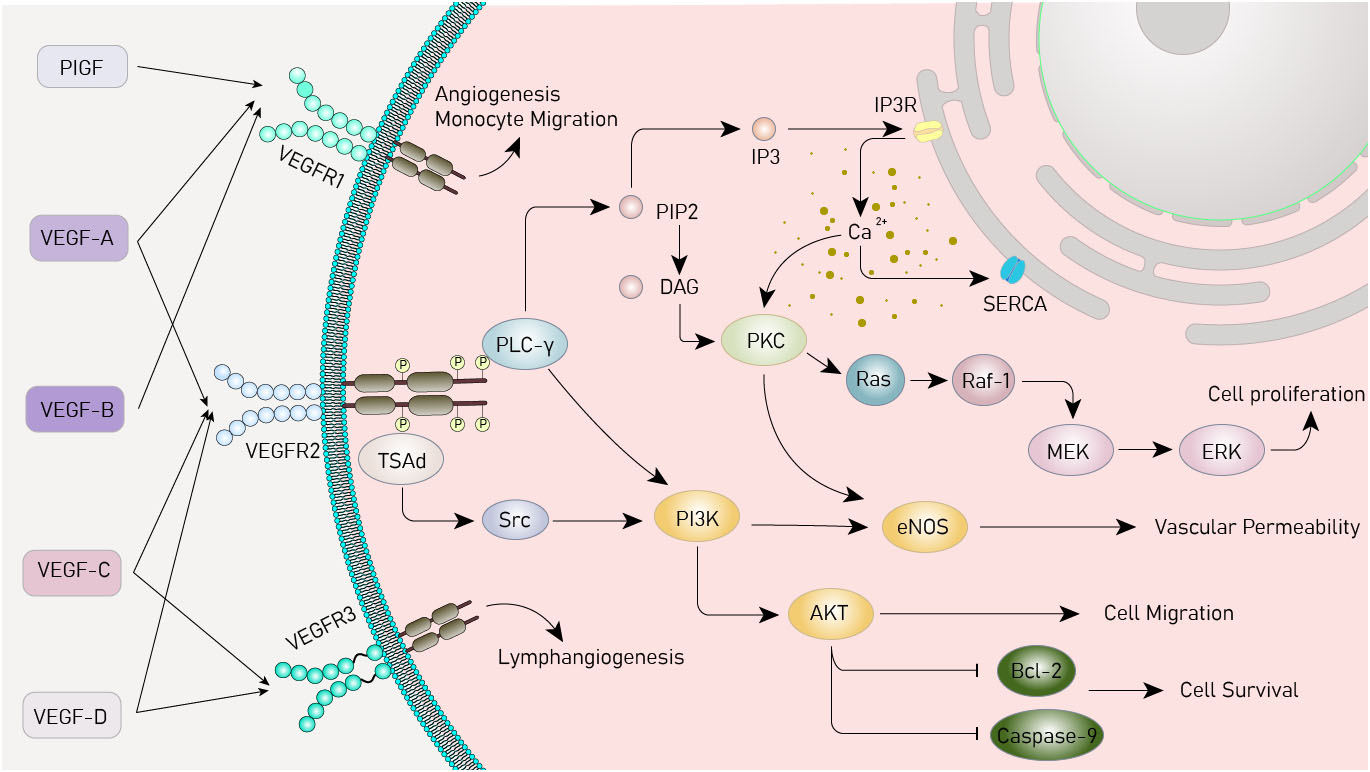
Vascular endothelial growth factor (VEGF) signaling pathway. VEGF, vascular endothelial growth factor; VEGFR, vascular endothelial growth factor receptor; PIGF, placental growth factor; PI3K, phosphoinositide 3-kinases; Akt, protein kinase B; MAPK, mitogen-activated protein kinase; PLC-γ, phospholipase C-γ; PIP2, phosphatidylinositol 4,5-bisphosphate; DAG, diacylglycerol; IP3, inositol trisphosphate; IP3R, inositol trisphosphate receptor; SERCA, sarcoplasmic/endoplasmic reticulum calcium ATPase; PKC, protein kinase C; Ras, rat sarcoma virus oncogene homolog; Raf-1, rapidly accelerated fibrosarcoma 1; ERK, extracellular signal - regulated kinase; TsAd, T cell-specific adapter protein; Src, sarcoma gene; eNOS, endothelial nitric oxide synthase; Bcl-2, B-cell lymphoma/leukemia -2.

The secreted glycoprotein family to which VEGF belongs contains six members: VEGF-B, -C, -D, -E, and placental-derived growth factor (PlGF). VEGF-A is the prototype member of this family. Among the four identified splicing variants of VEGF-A, VEGF120, which is early-active, and VEGF164, which is upregulated in adulthood, are the primary brain subtypes. The receptors that can specifically bind to VEGF are called vascular endothelial growth factor receptors (VEGFRs), including VEGFR-1 (Flt-1), VEGFR-2 (Flk-1), and VEGFR-3 (Flt-4). VEGFRs have expression specificity at different developmental stages and in different brain regions (de Vries et al., 1992; Licht and Keshet, 2013). In addition, NRP-1 and NRP-2 of the neuropilin family (NRP) can also bind to VEGF as co-receptors. Different members of VEGF bind to their specific receptors to exert various functions (Dakowicz et al., 2022). Binding VEGF-A, VEGF-B, and PlGF to VEGFR-1 can promote angiogenesis, hematopoiesis, and inflammatory cell recruitment. VEGFR-2 binds to VEGF-A with low affinity and can bind to proteolytically processed VEGF-C and VEGF-D, playing important roles in maintaining endothelial cell proliferation, blood-brain barrier permeability, neuronal cell survival and metastasis, and axon guidance. The binding of VEGFR-3 to VEGF-C and VEGF-D is a key regulatory factor for lymphatic endothelial function (Koch and Claesson-Welsh, 2012; Lange et al., 2016; Figure 1).

### 2.2 Signaling mechanisms of VEGF

Vascular endothelial growth factor plays an important physiological role by activating important downstream signaling pathways. The pathways include the PI3K/Akt, MAPK, and PLCγ. Activation of phosphatidylinositol 3-kinase (PI3K) by the VEGF receptor upregulates protein kinase B (Akt) protein, which inhibits apoptosis and promotes cell growth and survival (Jiang and Liu, 2008; Zachary, 2003) and the mitogen-activated protein kinase (MAPK) pathway involves a series of cascade reactions, which ultimately regulate the process of cell proliferation, migration, and survival and promote angiogenesis (Breslin et al., 2003; Estrada et al., 2019; Simons and Eichmann, 2015). In the phospholipase C-γ (PLCγ) pathway, activated PLCγ hydrolyzes phosphatidylinositol 4,5-bisphosphate (PIP2) on the cell membrane, generating the secondary messenger molecules diacylglycerol (DAG) and inositol 1,4,5-trisphosphate (IP3), and elevating the intracellular calcium ion concentration (Chen and Simons, 2021; Sjöberg et al., 2023; Figure 1).

In the human brain, VEGF promotes neurogenesis, and its roles in protecting neural function and synaptic plasticity also rely on the aforementioned mechanisms. Studies have demonstrated that catalpol, an extract of traditional Chinese medicine, exerts effects such as promoting cortical axonal growth, improving the neuronal microenvironment, and inhibiting neuronal apoptosis by enhancing VEGF-PI3K/AKT and VEGF-MAPK/ERK signaling (Wang et al., 2019, 2022). Similarly, the neuroprotective effects of danshensu, including improving cerebral blood flow in the peri-infarct region and alleviating behavioral and cognitive deficits in mice after middle cerebral artery infarction, have been confirmed to be achieved by upregulating VEGF levels through the regulation of the PI3K/AKT/mTOR pathway. Furthermore, VEGF deficiency may induce progressive neuronal degeneration, and reduced neurovascular perfusion accelerates the progression of neurodegeneration. *In vitro* experiments have verified that even under conditions of stress and susceptibility genes, VEGF can prevent the downregulation of B-cell lymphoma/leukemia-2 (Bcl-2), inhibit neuronal apoptosis, delay motor neuron degeneration, and improve neuronal survival by increasing PI3K and MAPK activity (Li et al., 2003; Lunn et al., 2009; Oosthuyse et al., 2001).

It can be seen from this that the functions of VEGF are not merely confined to traditional angiogenesis and the regulation of vascular permeability. It is also of great significance in neuronal protection. However, assessing VEGF content remains a research topic that needs further exploration. Since the human body is susceptible to VEGF, the constancy of its level is necessary for maintaining the functions of endothelial cells and the BBB. Pathologically, increased VEGF can lead to enhanced vascular permeability and leakage, damage the integrity of the BBB, and trigger the onset of various neurological diseases (Ruiz de Almodovar et al., 2009).

### 2.3 The relationship between VEGF and MDD

Numerous studies, through animal experiments and clinical trials, have confirmed from multiple perspectives that VEGF level deficiency is associated with the pathogenesis of depression. A plasma comparison study involving 40 MDD patients and 40 healthy controls showed that peripheral VEGF levels were negatively correlated with the severity of MDD (Kotan et al., 2012). A recent study covering 469 MDD cases further revealed that plasma VEGF levels in patients during the episode were significantly lower than those in healthy controls, and remained lower even after antidepressant treatment (Rigal et al., 2020). Additionally, other studies have confirmed that reduced VEGF levels also exist in the CNS of MDD patients (Kranaster et al., 2019). Suicide, as the most severe consequence of MDD, has an incidence of suicidal ideation as high as 37% among patients (Navarro et al., 2023). Clinical data indicated that cerebrospinal fluid VEGF levels in suicide attempters were significantly lower than those in the control group, and such levels were significantly negatively correlated with the severity of depression assessed by the MADRS scale, which is consistent with previous research conclusions (Isung et al., 2012a,2012b). Furthermore, chronic stress is an important factor in inducing and exacerbating MDD. An animal experiment measured the surface area covered by the vasculature, the proportion of vessel-associated newborn cells, and analyzed the expression of VEGF and Flk-1 proteins in the hippocampus of rats in the control group, chronic stress group, and recovery group. The results showed that 32% of proliferating cells in the rat hippocampus were associated with blood vessels. After chronic stress, the levels of VEGF and Flk-1 proteins in the granular cell layer were significantly decreased (Heine et al., 2005). Low VEGF levels may reflect insufficient neurotrophic support and reduced neurogenesis in the CNS, leading to atrophy of limbic structures that regulate emotions, impaired synaptic plasticity, and subsequent depressive symptoms (Clark-Raymond et al., 2014; Vaseghi et al., 2023). Peripheral and central VEGF are both relatively independent and interact through complex mechanisms such as the HPA axis. Therefore, reduced peripheral VEGF levels may be a consequence of decreased central VEGF, which to some extent reflects abnormal central neuroplasticity in MDD patients (Cao et al., 2005; Mikulska et al., 2021).

The restoration of VEGF levels is closely associated with the improvement of MDD symptoms. The VEGF and FIk-1 signaling pathways are important routes for antidepressant treatment. The latest animal experiments show that encapsulated mesenchymal stem cells (eMSCs) can effectively alleviate the depressive symptoms of treatment-resistant depressed rats. The antidepressant effect of eMSCs is related to its up-regulation of VEGF and Flk-1 expression in the hippocampus (Kin et al., 2020). Moreover, knocking out VEGF in the dentate gyrus of the hippocampus of mice using specific viruses can reduce hippocampal neurogenesis and induce depressive-like behaviors in specific behavioral tests (forced swimming, novel feeding), and these changes are partially blocked by ketamine (Choi et al., 2016). Different types of antidepressants have the effect of promoting hippocampal cell proliferation and neurogenesis, which is considered to be one of the bases of their antidepressant effects (Kodama et al., 2004; Malberg et al., 2000). VEGF is an important mediator of the neurogenic and behavioral effects of multiple types of antidepressants. Four experimental models of depressive behaviors have demonstrated that traditional antidepressants such as selective serotonin reuptake inhibitors (SSRIs) like fluoxetine, tricyclic antidepressants (TCAs) like desipramine, and even electroconvulsive therapy (ECT) can increase VEGF mRNA in the granule cell layer of the hippocampus. Selective, potent inhibitor experiments further clarify that VEGF/Flk-1 signal transduction is indispensable for antidepressant-induced cell proliferation (Deyama and Kaneda, 2023; Warner-Schmidt and Duman, 2007).

Interestingly, in recent years, multiple studies have examined the association between VEGF levels in humans and the severity of MDD from various perspectives, with results showing heterogeneity compared to previous data. For instance, a study investigating changes in plasma VEGF protein concentrations in adolescents with MDD after short-term antidepressant treatment revealed that VEGF protein levels were abnormally elevated in these adolescents, and antidepressant treatment could downregulate such levels (Krivosova et al., 2023). Another study, which compared serum VEGF levels between MDD patients and healthy subjects, observed significantly increased VEGF mRNA levels and serum protein concentrations in the MDD group (Berent et al., 2014; Maes et al., 2024). Additionally, clinical studies have confirmed that plasma VEGF levels in MDD patients rise during acute episodes (Lee and Kim, 2012). Research exploring changes in VEGF levels from the perspective of astrocyte-derived extracellular vesicles (ADEVs) also indicated that baseline VEGF levels in plasma ADEVs of MDD patients were elevated (Li et al., 2025). Maintaining a constant low level of VEGF is necessary: insufficient VEGF levels can affect the integrity of the brain, particularly the mesolimbic structures, while abnormally elevated VEGF levels can promote BBB permeability and excessive formation of new leaky blood vessels (Wu et al., 2022; Zhang et al., 2000). Both scenarios may occur in MDD patients, contributing to the pathogenesis and progression of the disease. The reasons for such discrepancies may be multifaceted. For example, in patients with acute MDD, stress triggers inflammatory responses and transient activation of the HPA axis, leading to a compensatory increase in VEGF to maintain neurovascular stability (Furtado and Katzman, 2015). Furthermore, obese patients may exhibit elevated peripheral VEGF due to leptin resistance (Caroleo et al., 2019). Patient-specific factors (age, gender), sample types (plasma, serum), and disease states (acute phase, medication status, specific subtypes, comorbidities) may all be important factors mediating such deviations (Homorogan et al., 2021; Kirschner et al., 2011). Therefore, in formulating VEGF-targeted therapeutic regimens for depression, clarifying the extent to which confounding factors affect VEGF levels in MDD patients may be a key direction for future research.

## 3 The mechanism of the association between VEGF and MDD

### 3.1 Gene

Major depressive disorder is a familial disease with a heritability of approximately 30%–40% (Sullivan et al., 2000). The genetic traits of MDD are complex and subtle, influenced by multiple variations (Ripke et al., 2013). A meta-analysis of data from 807,553 individuals across multiple MDD genome-wide association studies (GWAS) identified 102 independent variants associated with MDD, involving 269 genes and 15 gene sets (Howard et al., 2019). Genetic associations with MDD have also been observed for gender, height, weight, and comorbidities (Badamasi et al., 2019; Labonté et al., 2017; Lango Allen et al., 2010; Speliotes et al., 2010). The VEGF-related single-nucleotide polymorphism (SNP) rs69994 is associated with treatment-resistant depression (Viikki et al., 2010). As the role of VEGF in the pathological mechanism of MDD is gradually revealed, how SNPs in its gene and receptors affect depression susceptibility, symptom severity, and treatment response by regulating VEGF expression or function has become a research focus.

Firstly, VEGF-related SNPs are involved in MDD pathological mechanisms by regulating expression or function. For example, a variant at the rs4416670 locus of the VEGF gene has been confirmed to reduce VEGF transcriptional activity, leading to decreased expression levels, and individuals carrying this risk allele have a significantly increased risk of developing MDD (Xie et al., 2017). This finding is consistent with the core role of VEGF in maintaining neurovascular homeostasis–insufficient VEGF expression may disrupt vascular integrity in mesolimbic systems (such as the hippocampus), exacerbate neuronal damage, and thereby increase depression susceptibility. Notably, the serum heritability of the VEGF gene is as high as 50%, suggesting that such SNPs have a broad impact on the overall regulation of VEGF levels in the population (Debette et al., 2011). Another case-control study focusing on SNPs of VEGF, Flt1, and kinase insert domain receptor (KDR) showed that the Flt1-related SNP rs7993418 (a key VEGF receptor) was significantly associated with lower MDD symptom intensity (*p* = 0.003). At the same time, homozygous AA carriers of the rs699947 polymorphism had higher VEGF concentrations (*p* = 0.002) and an association with an increased number of suicide attempts (*p* = 0.041) (Nunes et al., 2022), indicating that SNPs may be involved in pathology by affecting VEGF downstream signaling or receptor function.

Furthermore, SNP-mediated abnormal VEGF function is associated with brain structural changes in MDD. Abnormal hippocampal structure (especially volume reduction) is one of the core pathological features of MDD. A genome-wide association study showed that drug-naive MDD patients carrying the VEGF gene rs6921438 locus had significantly smaller left subiculum volumes in the hippocampus compared to healthy controls (*p* = 0.039), and a multiple regression model confirmed a “Genotype–diagnosis interaction” at this locus (Nguyen et al., 2019). This finding is highly consistent with clinical and basic research results on MDD: as a key region for emotional regulation and memory processing, structural abnormalities of the hippocampal subiculum have been repeatedly confirmed to be closely related to depressive episodes in clinical trials, animal models, and autopsy studies (Rosoklija et al., 2000; Stockmeier et al., 2004; Watanabe et al., 2017; Willard et al., 2013). Therefore, VEGF-related SNPs may influence hippocampal development or repair by regulating VEGF expression, thereby promoting disease progression.

Interestingly, the function of VEGF is not independent; it interacts with SNPs of genes in classic depression-related pathways such as the serotonin (5-HT) system, collectively participating in the pathological regulation of MDD. 5-HT1A is one of the most abundant serotonin receptors in the human brain and has long been proven to act as a key factor in MDD involved in emotional regulation (Kaufman et al., 2016). Studies have shown that activation of the 5-HT1A gene can induce upregulated VEGF expression in the hippocampus, suggesting a genetic link between 5-HT1A and VEGF (Samaddar et al., 2015). A recent study analyzing 528 samples of Han Chinese MDD patients from northern China demonstrated that the interaction effect between VEGF (rs699947, rs833061, rs2010963) and 5-HT1A (rs6295, rs1364043, rs878567) genes has a clear pathological correlation with MDD (CV consistency = 10/10, *P* = 0.0107) (Han et al., 2019).

In addition, VEGF-related SNPs may also affect the therapeutic efficacy of MDD. Electroconvulsive therapy (ECT), which can increase hippocampal volume, promote neurogenesis, and enhance synaptic plasticity, has been proven to be the most effective biological therapy for MDD (Gbyl and Videbech, 2018; Kho et al., 2003; Olesen et al., 2017). A study based on elderly MDD patients combining MRI and genotyping observed that the rs699947 genotype could influence hippocampal volume after ECT, particularly in the right hippocampus (Van Den Bossche et al., 2019). Another study showed that alleles associated with lower VEGF concentrations blocked the therapeutic effect of ECT in MDD patients (*p* = 0.01), and this result was mediated by SNPs at the 6p21.1 gene locus (Maffioletti et al., 2020; Minelli et al., 2014). Additionally, the rapid antidepressant effect of ketamine has been shown to be associated with the VEGF rs79568085 genotype (Tsai et al., 2023).

Thus, VEGF-related SNPs are involved in MDD pathology by regulating VEGF expression (e.g., rs4416670, rs699947) or function (e.g., rs7993418). They promote disease occurrence by affecting hippocampal structure (e.g., rs6921438), amplify pathological effects through interactions with genes such as 5-HT1A, and can serve as biomarkers for predicting treatment responses to ECT, ketamine, etc. Future studies need to verify specific molecular mechanisms through functional experiments and expand sample sizes to clarify their roles in different populations, thereby promoting the translation from genetic associations to clinical applications.

### 3.2 Blood-brain barrier

The BBB is a semi-permeable and highly selective system that regulates the transport of substances in the brain and maintains the stability of the CNS environment. It separates the blood from the brain’s extracellular fluid and prevents neurotoxic substances, blood cells, and pathogens from entering the brain (Alahmari, 2021). Angiogenesis and blood vessel pattern formation driven by VEGF/Flk-1 signal transduction are significant for forming and fine-tuning the BBB (Wälchli et al., 2015). Abnormalities in the structure and function of the BBB can disrupt the brain’s environmental homeostasis and induce various risk factors such as inflammation, endothelial degeneration, capillary leakage, and protein deposition, thus establishing pathological connections with multiple neurological diseases (Sweeney et al., 2019).

A growing body of evidence indicates structural and functional impairments of the BBB in patients with MDD. First, S100B is a calcium-binding protein produced by glial cells and is widely used as a molecular marker for BBB disruption and brain injury (Gayger-Dias et al., 2023). A meta-analysis incorporating the results of 93 studies revealed that the blood of MDD patients contained elevated levels of S100B. Additionally, alterations were observed in other BBB protein markers, including fibrinogen, Aβ, and MMPs. These changes indicated an augmentation in BBB leakage (Futtrup et al., 2020). The tight-junction protein claudin-5 related to the BBB has also been proven to have decreased expression in the hippocampus of MDD individuals. Other tight-junction mRNA transcripts (claudin-12 and ZO-1) are associated with the age of onset and duration of MDD (Greene et al., 2020). Moreover, a recent study recruited 23 MDD patients and 18 healthy subjects. It calculated the mean volume transfer constant (Ktrans) through dynamic contrast-enhanced magnetic resonance imaging (DCE-MRI) to assess the BBB leakage status. The results showed that the Ktrans in the local brain regions of MDD patients was elevated and decreased after treatment. Moreover, the Hamilton Anxiety Scale (HAMA) score was positively correlated with Ktrans, suggesting that BBB leakage is significantly correlated with MDD and depressive symptoms (Shang et al., 2024). Finally, stress is a recognized key factor leading to MDD. Animal experiments have confirmed that chronic stress can disrupt the BBB structure in the emotion-related brain regions of female mice and induce anxiety and depressive-like behaviors (Dion-Albert et al., 2022).

**FIGURE 2:**
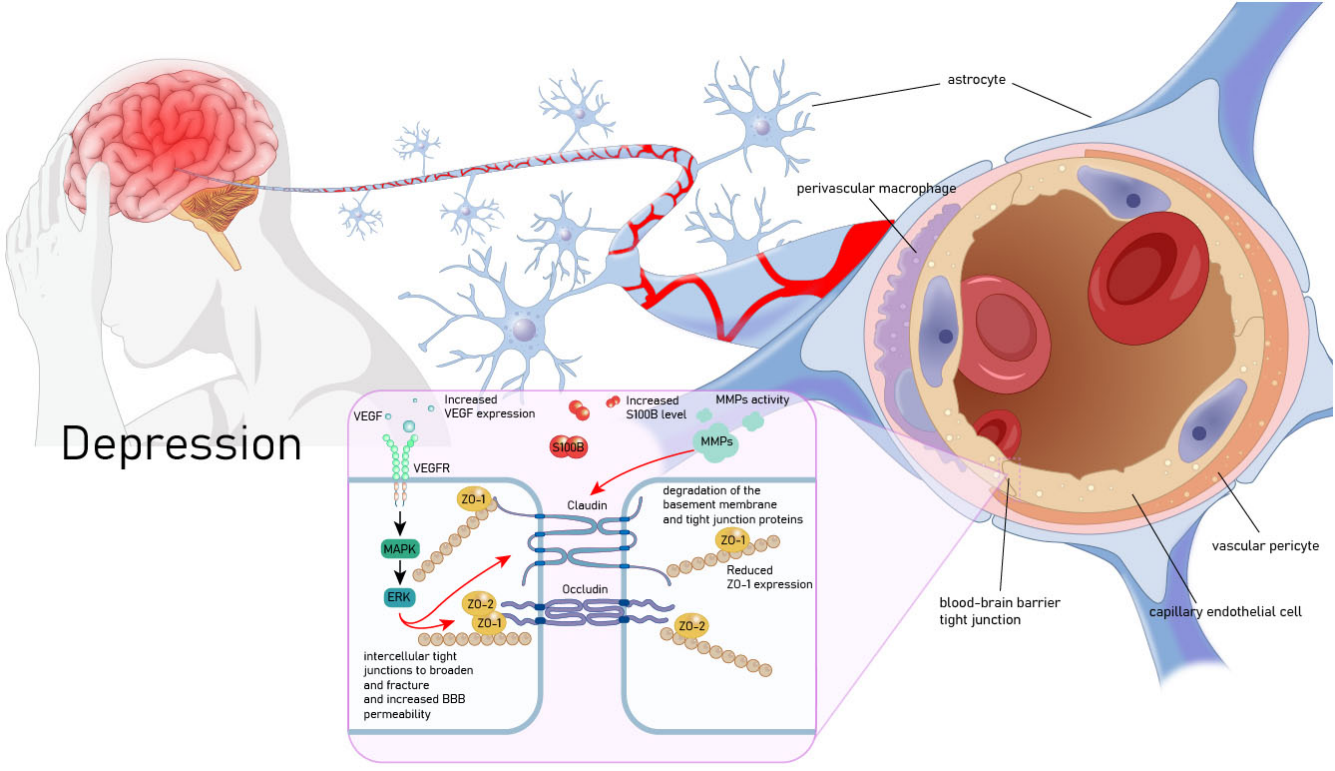
Vascular endothelial growth factor (VEGF) mediates the pathological association between MDD and BBB. VEGF, vascular endothelial growth factor; VEGFR, vascular endothelial growth factor receptor; MAPK, mitogen-activated protein kinase; ERK, extracellular signal - regulated kinase; ZO-1, zonula occludens - 1; ZO-2, zonula occludens - 2; MMPs, matrix metalloproteinases; S100B, S100 calcium - binding protein B.

Vascular endothelial growth factor may mediate the link between MDD and BBB dysfunction. Firstly, astrocytes have a unique structure that enwraps endothelial cells and releases neurotrophic factors to maintain the BBB (Zidarič et al., 2022). Consequently, VEGF released by astrocytes regulates BBB permeability (Manu et al., 2023). Further research has confirmed that the up-regulation of VEGF levels induced by the activation of astrocytes in MDD patients significantly increases the permeability of the BBB, which enables more pro-inflammatory cytokines to enter the brain, fuel neuroinflammation in the human brain, and leads to depressive-like behaviors (Argaw et al., 2009; Dudek et al., 2020). Moreover, the VEGF/Flk-1 pathway may be an important molecular route through which BBB breakdown induces depressive behaviors. Research shows that microwave radiation can induce changes in BBB permeability by activating the VEGF/Flk-1 - ERK pathway and down-regulating the expression of BBB - related proteins occludin and zonula occludens-1 (ZO-1) (Wang et al., 2015). Conversely, inhibiting the expression of Flk-1 using specific monoclonal antibodies can effectively restore the BBB permeability in the chronic restraint stress (RS) animal model and alleviate various depressive behaviors (Matsuno et al., 2022; Figure 2).

All these findings indicate a key pathological link between MDD and BBB dysfunction, with VEGF potentially mediating this relationship. The contradiction between its vascular permeability-disrupting effects and neuroprotective roles can be explained by temporal dynamics and receptor selectivity: transient elevation of VEGF during acute stress exerts protective effects by promoting angiogenesis and supporting neurotrophy, whereas sustained high expression during the chronic depressive phase leads to BBB leakage, enabling peripheral inflammatory factors to infiltrate and damage neurons (Kaya et al., 2005; Proescholdt et al., 1999). Furthermore, the VEGF receptors Flt-1 and Flk-1 exert antagonistic functions in MDD: Flt-1 disrupts BBB tight junctions (e.g., ZO-1, occludin) via the PLCγ/NO pathway, mediating pathological permeability; in contrast, Flk-1 promotes neuronal survival and synaptic plasticity through the PI3K/Akt pathway (Mesquita-Britto et al., 2020; Wang et al., 2024). Such differences in receptor selectivity determine the disease trajectory. Thus, exploring individual variations and identifying universal principles for maintaining the low-permeability homeostasis of the BBB may represent a potential direction for MDD treatment, though more direct evidence is still required to support this.

### 3.3 Brain-derived neurotrophic factor

The neurotrophic hypothesis of MDD posits that reduced neurotrophic factors, especially brain-derived neurotrophic factor (BDNF) and VEGF, can induce neuronal atrophy in brain regions associated with MDD, an important mechanism underlying depressive symptoms (Duman et al., 2016; Rana et al., 2021; Uslu and Yildiz, 2024). BDNF is the most highly expressed member of the nerve growth factor family. It contributes to the survival, growth, and maintenance of neurons and is involved in various learning and memory-related functions, playing a crucial role in regulating activity-dependent neuronal plasticity (Ateaque et al., 2023). The specific receptor tyrosine kinase receptor (TrkB) is an important high-affinity receptor for BDNF. BDNF must bind to it to activate different downstream signaling pathways (such as PI3K/Akt, Raf/ERK) and exert various neurotrophic effects (Zeng et al., 2024). For example, recent results have shown that by up-regulating the BDNF/TrkB - mediated PI3K/Akt/mTOR signaling pathway, the expression of postsynaptic density 95 and synapsin in the hippocampus of MDD rats can be increased, and the depressive behaviors of the lipopolysaccharide (LPS) -induced MDD rat model can be improved (Wu et al., 2024).

However, there is an interrelated effect between BDNF and VEGF. On the one hand, VEGF has the capacity to upregulate the production of BDNF. Research has found that the knockout of Flk - 1 accelerates the reduction of BDNF (Le et al., 2021). Moreover, single-nucleus RNA sequencing has revealed that VEGF exerts a neuro-supportive function by upregulating the BDNF signaling pathway in brain cells and reducing ischemic stroke damage (Simoes Braga Boisserand et al., 2023). On the other hand, BDNF can also promote an increase in VEGF secretion. The VEGF mRNA levels in neuroblastoma cells rise 8–16 h after exposure to BDNF. BDNF induces a 2- to 4-fold increase in VEGF promoter activity (Nakamura et al., 2006). Another study has confirmed that BDNF can stimulate the expression and secretion of VEGF in osteoblasts through the TrkB/ERK signaling pathway (Zhang et al., 2017).

**FIGURE 3:**
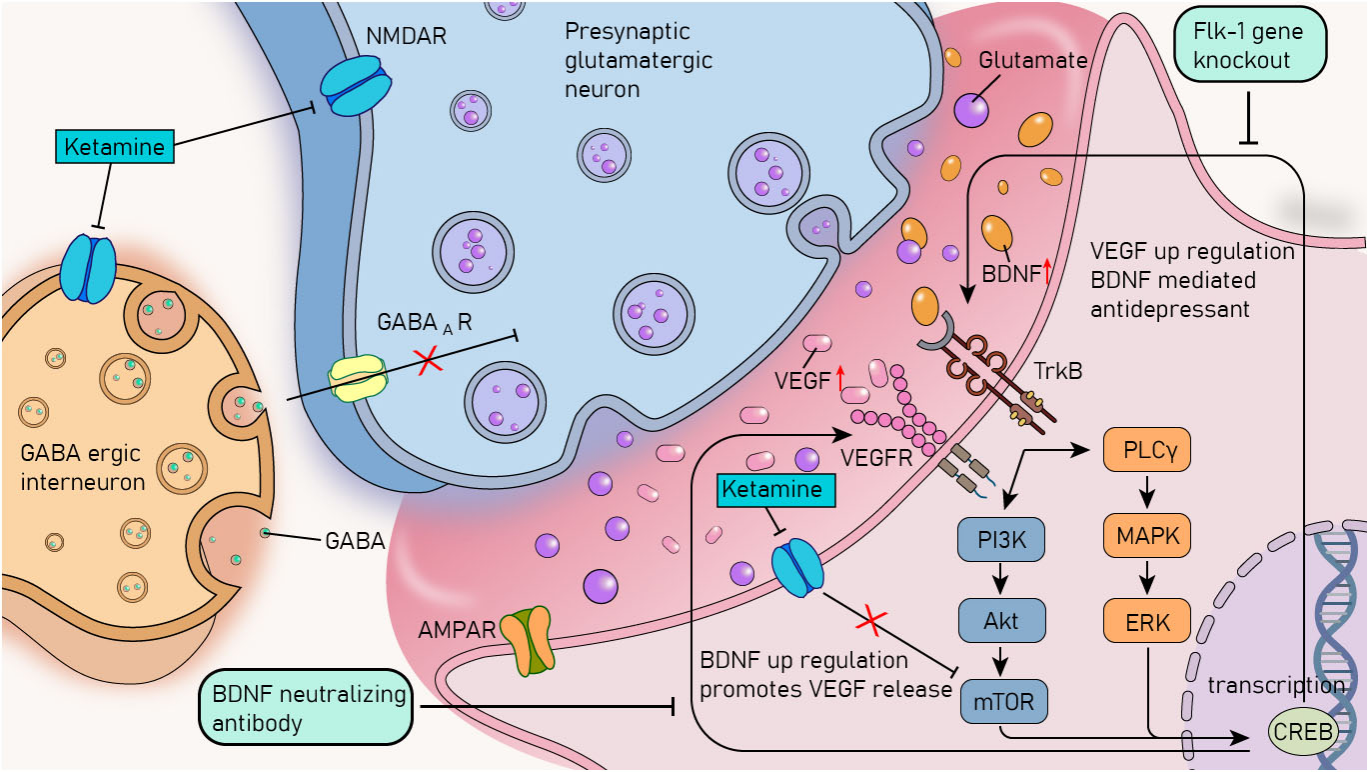
The crosstalk mechanism between BDNF and VEGF and the rapid antidepressant mechanism of ketamine. VEGF, vascular endothelial growth factor; BDNF, brain - derived neurotrophic factor; TrkB, tyrosine kinase receptor B; VEGFR, vascular endothelial growth factor receptor; PI3K, phosphoinositide 3-kinases; Akt, protein kinase B; MAPK, mitogen-activated protein kinase; PLCγ, phospholipase Cγ; ERK, extracellular signal - regulated kinase; mTOR, mammalian target of rapamycin; GABA, γ - aminobutyric acid; GABAAR, γ - aminobutyric acid type A receptor; CREB, cAMP - responsive element binding protein; NMDAR, N - methyl - D - aspartate receptor; AMPAR, α - amino - 3 - hydroxy - 5 - methyl - 4 - isoxazolepropionic acid receptor.

In the pathological process of MDD, there is also an interdependence between BDNF and VEGF. The neuroprotective and antidepressant-like effects of BDNF and VEGF necessitate close collaboration between them (Dattilo et al., 2020). On the one hand, the antidepressant - effects of BDNF in three different behavioral paradigms (behavioral despair model, motivation and rewards model, and anxiety model) rely on neuron-derived extracellular VEGF support. Moreover, the influence of BDNF-induced neurotrophic effects on dendritic complexity requires VEGF/Flk-1 signaling in primary cortical neurons. On the other hand, BDNF/TrkB signal transduction promotes the release of VEGF in neurons. In the medial prefrontal cortex (mPFC), the co-infusion of BDNF-neutralizing antibodies can block the antidepressant behavior induced by VEGF infusion (Deyama et al., 2019; Figure 3).

The crosstalk between neurotrophic factors also plays a crucial role in treating MDD. For example, research has found that the increased expression of these factors can restore the brain’s structural and functional impairments caused by stress and depression. It provides trophic support and protection, thereby reversing the atrophy and synaptic loss that lead to the pathophysiology of MDD, and mediates the rapid antidepressant effect of ketamine (Duman et al., 2021; Joshi and Salton, 2022; Figure 3). Moreover, the latest research has confirmed that local infusion of resolvin E1 (RvE1) into the mPFC or the dorsal hippocampal dentate gyrus (DG) through intracerebroventricular infusion can upregulate mTOR by activating the activity-dependent BDNF/VEGF pathway, thus producing antidepressant-like effects (Deyama et al., 2023).

These findings highlight the synergistic effect of BDNF and VEGF in the physiological and pathological processes of MDD. The inhibition of BDNF and VEGF can block the antidepressant effect of a single neurotrophic factor. Targeted treatment of MDD by targeting BDNF and VEGF may be a new research direction in the future.

## 4 Antidepressant treatment with VEGF as the therapeutic target

### 4.1 Traditional antidepressants

Classic antidepressants, such as tricyclic antidepressants (TCAs) and selective serotonin (5 - HT) reuptake inhibitors (SSRIs), have been shown to be capable of regulating VEGF and its related pathways. Preclinical data have shown that treatment with duloxetine at 20 mg/kg can increase plasma 5-HT levels and activate the PI3K/AKT/VEGF signaling pathway, thereby exerting a protective effect against indomethacin-induced gastric ulcers (Ding et al., 2024). Moreover, fluoxetine has also been proven to increase the protein expression of VEGF and its receptor VEGFR in the brain of rats with the middle cerebral artery occlusion model, promote angiogenesis, and play a protective role in neurological impairment after ischemic stroke (Hu et al., 2020).

Hippocampal neurogenesis plays a vital role in the antidepressant effects of traditional antidepressants, in which VEGF may play an important role. Early animal experiments demonstrated that long-term use of fluoxetine could increase VEGF mRNA in hippocampal neurons and endothelial cells. Meanwhile, the pharmacological inhibition of the VEGF/Flk-1 pathway blocked the improvement effect of fluoxetine in three behavioral tests (sucrose preference, forced swimming, and novelty suppression) in rats of the chronic unpredictable mild stress (CUMS) model (Deyama and Duman, 2020; Greene et al., 2009). A recent rodent experiment also proved that the antidepressant-like effects of repeated desipramine and chronic fluoxetine treatment could be entirely blocked by neuron-specific VEGF or Flk-1 knockout (Deyama et al., 2024a). However, these results are also controversial. For example, Chen’s research showed that vortioxetine could upregulate the protein level of VEGF in the hippocampus and improve the hippocampal microvasculature. In contrast, fluoxetine failed to improve these indicators (Chen et al., 2021).

### 4.2 Ketamine

Traditional antidepressants can rapidly increase extracellular monoamine levels, yet their clinical efficacy is slow to manifest, often taking weeks to months. This delay in effectiveness increases the risk of suicidal behavior during the first month of antidepressant treatment (Jick et al., 2004). Meanwhile, the efficacy of monoaminergic antidepressants is limited, with approximately one-third of patients showing no response to the drugs (Trivedi et al., 2006). Therefore, there is an urgent need to break through the mechanisms of traditional antidepressants and pursue more efficient and rapid-acting antidepressant treatments.

Ketamine is a non-competitive N - methyl - D - aspartate receptor (NMDAR) antagonist that has been used as a clinical anesthetic for over half a century (Lavender et al., 2020). Subsequently, a series of studies have uncovered that ketamine has rapid (within a few hours) and long-lasting (up to a week) antidepressant effects, making it a promising new - type of antidepressant for treating treatment-resistant depression and various mixed mood disorders (Berman et al., 2000; Zarate et al., 2006). The molecular mechanisms of ketamine’s antidepressant effects are extensive. Increasing central neurogenesis and improving synaptic plasticity are among its core features, which are closely related to its ability to promote the release of neurotrophic factors (BDNF, VEGF) (Kim et al., 2024; Figure 3). Rodent experiments have confirmed that VEGF and its signaling pathway influence the antidepressant effect of ketamine. Deyama et al. (2024a) have fully elucidated the potential mechanism of the relationship between VEGF/Flk-1 signal transduction and the rapid antidepressant effect of ketamine through a series of co - infusion experiments of neutralizing antibodies and behavioral methods. Neuron-specific deletion of VEGF or Flk-1 in the mPFC and hippocampus can effectively block the antidepressant effect of ketamine. Infusing anti-VEGF neutralizing antibodies into the mPFC 30 min before ketamine administration blocks the antidepressant effect of ketamine, and in contrast, infusing the same antibody 2 h after ketamine administration does not affect the drug’s efficacy (Kenwood et al., 2019; Figure 3). A clinical study involving 25 patients with MDD examined plasma samples collected 1 h before and 4 h after ketamine infusion at a dosage of 0.5 mg/kg. The results of this study confirmed that a single infusion of ketamine can lead to an increase in the level of VEGF. Moreover, a significant group × time interaction was observed in the mRNA level of VEGF, with a *p*-value of 0.029 (McGrory et al., 2020).

However, the research findings regarding the association between VEGF and the antidepressant effect of ketamine are inconsistent. A study conducted six infusions of ketamine (0.5 mg/kg) on 78 MDD patients and found that, compared with the baseline, there was no difference in the change of plasma VEGF concentration between anhedonia responders and non-responders on the 13th and 26th days of treatment (*p* > 0.05) (Zheng et al., 2021). Another clinical study involving 48 MDD patients also confirmed that a single infusion of 0.5 mg/kg ketamine did not alter the VEGF level in MDD patients (Chen et al., 2023).

This discrepancy may arise from multiple factors. Firstly, VEGF in peripheral plasma may not timely reflect its expression in the brain. Secondly, various factors in clinical studies (such as age, gender, treatment course, and administration time) may affect the detection results. Further research is required to explore the impact of VEGF on the rapid and long-term antidepressant effects of ketamine.

### 4.3 Electroconvulsive therapy

The efficacy of ECT in treating MDD is closely associated with its induction of neuroplastic changes, in which VEGF plays a critical mediating role (Maffioletti et al., 2021). Accumulating evidence indicates that ECT can rapidly and transiently increase VEGF levels in both the brain and peripheral circulation, and this dynamic change is considered an initiating mechanism for ECT-triggered adaptive neuroplastic remodeling.

Clinical studies have demonstrated the responsive pattern of VEGF to ECT stimulation. A study involving 110 MDD patients confirmed that 8-weeks treatment with combined ECT and medication significantly upregulated serum VEGF levels. In contrast, no such effect was observed in the medication-only group, reflecting the specific regulatory role of ECT on VEGF (Zhang et al., 2023). Dynamic monitoring at specific time points after single treatments further revealed that plasma VEGF concentrations in patients increased significantly at 2 and 4 h after the first and fifth ECT sessions. Notably, despite the acute elevation of VEGF induced by single treatments, VEGF levels typically return to baseline after the completion of the entire ECT course, indicating the prominently short-term and transient nature of its upregulation (Sorri et al., 2021).

Animal experimental models provide deeper insights into the temporal profile of VEGF and its causal relationship with neuroplasticity. Rodent studies have confirmed that hippocampal VEGF levels increase rapidly within 3 h after ECT intervention and begin to decrease after 6 h, which is highly consistent with the rapid and transient pattern observed in clinical settings (Elfving and Wegener, 2012). More importantly, within 24 h after ECT treatment, significant increases in VEGF levels in the CA1, CA2, CA3, and DG regions of the hippocampus in MDD model rats were observed, accompanied by rapid improvements in hippocampal structural plasticity and synaptic ultrastructural plasticity (Chen et al., 2020). This suggests a temporal correlation between the acute elevation of VEGF and early beneficial remodeling of neuroplasticity.

Although VEGF levels may eventually return to baseline after repeated ECT sessions (Chen et al., 2020), the beneficial neuroplastic changes it triggers (such as a significant increase in hippocampal volume) can persist for at least 3 months (Nordanskog et al., 2010; Takamiya et al., 2023). This phenomenon suggests that the short-term significant rise in VEGF constitutes a critical initiating event for adaptive neuroplastic remodeling. Its role may lie in rapidly activating downstream signaling pathways, inducing processes such as synaptic protein synthesis, dendritic spine formation, or neurogenesis, thereby laying the foundation for long-term functional and structural changes in neural circuits (Madsen et al., 2000). Even after VEGF concentrations subsequently decline, the molecular and cellular cascades it initiates can persist, ultimately leading to a relatively stable neuroplastic state associated with symptom remission.

In summary, ECT induces a rapid and transient significant increase in VEGF, which serves as the initial driving force for initiating beneficial neuroplastic remodeling in the hippocampus and related brain regions. Although the acute changes in VEGF are time-limited, the biological effects it triggers are sufficient to promote long-term structural and functional optimization of neural circuits, providing an important molecular biological basis for understanding the antidepressant mechanism of ECT.

### 4.4 Repetitive transcranial electrical stimulation

Repetitive transcranial magnetic stimulation (rTMS) is a non-invasive brain stimulation technique that can serve as an adjunctive treatment for patients with MDD who respond poorly to antidepressant medications. Compared with ECT, rTMS does not require general anesthesia, has higher safety, fewer side effects, and better clinical acceptance (Kaster and Blumberger, 2024). Recent clinical studies have further indicated that rTMS may improve neuroplasticity by upregulating VEGF levels in the CNS, providing important clues for its antidepressant mechanism. For example, a randomized controlled trial by Xu B. et al. (2024) on patients with post-stroke cognitive impairment found that 4-weeks dual-target rTMS stimulation significantly increased serum VEGF levels, and the degree of VEGF elevation was positively correlated with improvements in cognitive function. Furthermore, a mechanistic study by Xing et al.’s (2023) in an animal model of ischemic stroke further revealed that rTMS treatment could significantly enhance VEGF expression in brain tissues, activate vasculogenesis and angiogenesis pathways, and help reverse neuronal death and synaptic structural damage associated with post-stroke dysfunction. Inspired by the findings of ECT studies, in recent years, many researchers have begun to explore whether VEGF is involved in the antidepressant mechanism of rTMS (Fukuda et al., 2020).

A large number of studies have confirmed the effectiveness of rTMS in improving neuroplasticity in MDD patients. The latest clinical study, using measurements of neurophysiological parameters of transcranial magnetic stimulation, confirmed that the baseline level of Long-Term Potentiation-like (LTP-like) plasticity in the motor cortex of MDD patients is low, and the improvement of clinical symptoms after rTMS intervention is positively correlated with the increase in LTP-like plasticity (Scho et al., 2024). A clinical study based on resting-state functional connectivity further showed that the enhancement of neural functional connectivity between the rTMS stimulation site and the limbic network is a key mechanism mediating antidepressant efficacy (Long et al., 2024). Several recent clinical and preclinical studies have suggested that VEGF may be involved in the mechanism by which rTMS induces neurogenesis and remodels synaptic plasticity to exert antidepressant effects. A study by Elemery et al. (2022) recruited 17 patients with clinically refractory depression who received 10 sessions of medication combined with rTMS treatment, and found that serum VEGF is sensitive to changes in anhedonia during rTMS treatment, and baseline VEGF levels may be an important predictor of rTMS treatment efficacy. Xu Q. et al. (2024) found that mice exposed to a simulated spatially complex environment (SSCE) for 7 days exhibited depression-like behaviors, while 14-days rTMS treatment could significantly improve SSCE-induced emotional and social dysfunction by regulating VEGF signaling in the mPFC.

These findings suggest that rTMS may exert neuroprotective effects by increasing VEGF levels, thereby playing a role in the treatment of depression. Meanwhile, baseline VEGF levels may predict the therapeutic effect of rTMS. However, the sample sizes of the studies and the number of conducted studies are small, requiring further data for confirmation.

### 4.5 Resolvins

The resolvin D series (RvD1, RvD2) and E series (RvE1, RvE2, RvE3) are bioactive lipid mediators derived from docosahexaenoic acid and eicosapentaenoic acid, respectively, named for their critical role in the resolution of inflammation. As early as 2014, studies confirmed that RvD1 and RvD2 could ameliorate depressive-like behaviors in experimental animals (Gilbert et al., 2014; Klein et al., 2014). Deyama (2020), through a series of experiments, demonstrated that exogenously administered resolvin could accelerate spontaneous recovery and mitigate depressive symptoms. For instance, the quantitative infusion of RvD1, RvD2, RvE1, RvE2, and RvE3 into the DG and mPFC could effectively improve the depressive behaviors of mice induced by LPS and CUMS, significantly reducing the immobility time in the tail-suspension test and forced-swimming test (Deyama et al., 2017, 2018a,Deyama et al., 2018b; Ishikawa et al., 2017). Secondly, large-scale clinical data analysis showed that treatment with synthetic glucocorticoids (e.g., prednisolone) increases the risk of MDD by 3-fold and the risk of suicide by 7-fold in patients (Fardet et al., 2012; Patten, 2000). A recent experiment on rodents has proven that a single dose of RvE1 can induce rapid and sustained neuroplastic changes in brain regions like the mPFC, reversing the increased immobility in the tail-suspension test induced by prednisolone (Aoki et al., 2022).

Animal studies have confirmed that the rapid antidepressant effect of RvE1 is closely associated with VEGF. At the molecular level, subcutaneous injection of resolvins can significantly upregulate VEGF gene expression in the rat brain and promote neovascularization (Jiang et al., 2022). Behavioral experiments further confirmed that intranasal administration of RvE1 exerts rapid and sustained antidepressant effects similar to those of ketamine (Deyama, 2020). Intranasal administration of RvE1 in LPS-induced depressive mice effectively reduced immobility in the tail suspension test. It forced a swimming test, a phenomenon that was entirely blocked by quantitative intracerebroventricular injection of VEGF-neutralizing antibodies (Deyama et al., 2024b). Thus, the antidepressant effect of RvE1 depends on VEGF released from the mPFC, similar to the mechanism of ketamine.

Resolvin has the potential to be a highly promising candidate drug for rapid-acting antidepressants in clinical practice. However, more research is needed to confirm its mechanism, efficacy, and safety.

## 5 Conclusion

The studies described in this review indicate that VEGF holds significant importance in the pathological process of MDD. VEGF may be pathologically intertwined with MDD through pathways involving genes, the BBB, and BDNF. At the same time, VEGF and its related pathways have also been shown to mediate numerous antidepressant treatments, such as traditional antidepressants, ketamine, ECT, rTMS, and resolvins. VEGF therapy may confer benefits in MDD by reducing depression susceptibility through enhancing vascular and neuroplasticity, as well as exerting direct neuroprotective effects. However, upregulating VEGF levels may trigger BBB disruption and vascular leakage. Existing evidence suggests that short-term rapid upregulation of VEGF expression in the CNS may trigger long-term molecular cascades, promoting progressive and beneficial remodeling of the neurovascular network, and the subsequent return of VEGF to baseline levels during treatment can avoid BBB damage. This mechanism is expected to become a core breakthrough point in the treatment of rapid-acting and treatment-resistant depression, with excellent research value.

Future research should focus on the differential mechanisms in specific populations (age/gender/ethnicity), disease phases (acute/chronic), brain region structures, and specific receptors (Flk-1/Flt-1). It is necessary to develop brain region-targeted delivery systems combining Flt-1 inhibitors and neuroprotective VEGF subtypes to promote angiogenesis and the recovery of neuroplasticity in the CNS while preventing BBB leakage. In-depth clarification of the physiological functions of VEGF in brain development will provide important support for developing novel antidepressant strategies and advancing the field of nervous system repair.
